# Quality-by-Design R&D of a Novel Nanozyme-Based Sensor for Saliva Antioxidant Capacity Evaluation

**DOI:** 10.3390/antiox12051120

**Published:** 2023-05-18

**Authors:** Riccardo Goldoni, Douglas Vieira Thomaz, Lucanos Strambini, Margherita Tumedei, Paola Dongiovanni, Gaetano Isola, Gianluca Tartaglia

**Affiliations:** 1 Department of Electronics, Information and Bioengineering (DEIB), Politecnico Di Milano, 20133 Milan, Italy; 2 CNR-Istituto di Elettronica e di Ingegneria dell’Informazione e delle Telecomunicazioni, 56122 Pisa, Italy; lucanos.strambini@ieiit.cnr.it; 3 National Enterprise for NanoScience and NanoTechnology (NEST), Istituto Nanoscienze-CNR and Scuola Normale Superiore, Piazza San Silvestro 12, 56127 Pisa, Italy; douglas.vieirathomaz@sns.it; 4 Department of Biomedical, Surgical and Dental Sciences, School of Dentistry, University of Milan, 20100 Milan, Italy; margherita.tumedei@unimi.it; 5 UOC Maxillo-Facial Surgery and Dentistry Fondazione IRCCS Cà Granda, Ospedale Maggiore Policlinico, 20122 Milan, Italy; 6 Medicine and Metabolic Diseases Fondazione IRCCS Cà Granda, Ospedale Maggiore Policlinico, 20122 Milan, Italy; paola.dongiovanni@policlinico.mi.it; 7 Department of General Surgery and Surgical-Medical Specialties, School of Dentistry, University of Catania, 95124 Catania, Italy; gaetano.isola@unict.it

**Keywords:** total antioxidant capacity, quality by design, nanozyme, electrochemical sensor, saliva

## Abstract

Oxidative stress is one of the main causes of cell damage, leading to the onset of several diseases, and antioxidants represent a barrier against the production of reactive species. Saliva is receiving increasing interest as a promising biofluid to study the onset of diseases and assess the overall health status of an individual. The antioxidant capacity of saliva can be a useful indicator of the health status of the oral cavity, and it is nowadays evaluated mainly through spectroscopic methods that rely on benchtop machines and liquid reagents. We developed a low-cost screen-printed sensor based on cerium oxide nanoparticles that can be used to assess the antioxidant capacity of biofluids as an alternative to traditional methods. The sensor development process was investigated via a quality-by-design approach to identify the most critical parameters of the process for further optimization. The sensor was tested in the detection of ascorbic acid, which is used as an equivalent in the assessment of overall antioxidant capacity. The LoDs ranged from 0.1147 to 0.3528 mM, while the recoveries varied from 80% to 121.1%, being therefore comparable with those of the golden standard SAT test, whose recovery value was 96.3%. Therefore, the sensor achieved a satisfactory sensitivity and linearity in the range of clinical interest for saliva and was validated against the state-of-the-art equipment for antioxidant capacity evaluation.

## 1. Introduction

Antioxidants are molecules that contribute to protect the body from oxidative stress and the damage caused by free radicals [1]. Oxidative stress (OS) results from an imbalance between reactive oxygen species (ROS) production and the endogenous antioxidant defense system [2]. ROS are produced as a byproduct of cellular metabolism and can cause oxidative damage to proteins, lipids, and DNA, leading to various diseases such as cancer [3], cardiovascular disease [4], and neurodegenerative disorders [5]. Therefore, monitoring antioxidant levels in the human body is of great importance for maintaining good health.

Antioxidant capacity (AOC), also referred to as antioxidant power or antioxidant activity, refers to the ability of an antioxidant species to neutralize free radicals and prevent oxidative damage to biological molecules [6,7]. Antioxidants can be endogenous, which means they are produced by our organism such as glutathione, superoxide dismutase, and catalase, or exogenous, which means they need to be obtained from external sources, such as vitamins C and E, carotenoids, and polyphenols. As biofluids contain several antioxidants of both endogenous and exogenous nature, total antioxidant capacity (TAC) has been introduced as a measure of the overall ability of a biological sample, such as blood or saliva [8], to scavenge free radicals through the cumulative action of all the antioxidants that are present in the sample [9].

Saliva is a promising biofluid for antioxidant analysis due to several reasons [10,11,12,13]. First, saliva is a non-invasive and easily accessible biological fluid that can be collected without the need for specialized equipment or medical personnel. Unlike blood, which requires invasive procedures, saliva can be collected by simple suction or spitting into dedicated saliva collection kits. This makes saliva collection more convenient and the workflow less stressful for patients. Third, saliva samples can be easily transported and stored without the need for specialized equipment or preservation methods. Therefore, saliva collection and processing are relatively inexpensive and can be easily scaled up for large-scale screening, making saliva a promising biofluid for diagnostic applications and screening of large cohorts [14,15,16]. Concurrently, advances in microelectronics and electrochemical readout systems [17] have allowed for a substantial reduction of the form factor of final devices, enabling the realization of point-of-care systems for rapid diagnostics.

In addition to its role in overall health, TAC is important for maintaining healthy oral tissues and preventing oral diseases in the context of oral health [18,19]. The oral cavity is a complex environment that is constantly exposed to various microorganisms, food debris, and other environmental factors. The production of ROS by bacteria in the oral cavity can lead to oxidative damage to oral tissues, including the gums, teeth, and tongue. Saliva contains various antioxidants that are crucial for maintaining oral health, such as uric acid, ascorbic acid, and glutathione. These antioxidants can neutralize free radicals produced during oral processes, such as chewing and swallowing, as well as during bacterial infections. Therefore, monitoring TAC in saliva can provide an indication of the overall antioxidant status of the oral cavity and its ability to protect against oxidative stress and damage. Low TAC values in saliva have been associated with an increased risk of the development and progression of various oral diseases [20], such as periodontitis [21], caries [22], and oral cancer [23]. For example, a study found that patients with periodontitis had significantly lower TAC values in their saliva compared with healthy controls [24]. Similarly, a study found that patients with oral cancer had significantly lower TAC values in their saliva compared with healthy controls [25].

Therefore, monitoring TAC in saliva can provide a valuable tool for the early detection and prevention of oral diseases, ultimately improving oral health and overall well-being. Various analytical methods [26] have been developed to measure TAC in biological samples, including spectrophotometry, chromatography, and electrochemistry. One such test is the SAT test, which is based on a spectrophotometric principle and has been clinically validated for screening of periodontal disease [27]. SAT stands for Salivary Antioxidant Capacity test and relies on the color change promoted by the reduction of ferric ion to ferrous ion in the presence of a thiocyanate-based chromogen. The miniSAT (Figure S1) is a dedicated spectrophotometer that is used to detect the color change and provide a measurement of the antioxidant capacity of the sample. The test became a golden standard in odontology due to its easiness of use, brief execution time, and robustness, as it is less affected by influences from salivary phosphates. Moreover, due to its portable nature, it can be readily used in a point-of-need setting, such as during patient visits to dental practices. Electrochemical sensors, however, have emerged as a promising tool for the detection of antioxidants due to their high sensitivity, selectivity, low cost, and real-time monitoring capability [28]. These sensors can be based on a variety of voltammetric and amperometric techniques, aiming at correlating an electrical response with the total antioxidant capacity or the concentration of a specific antioxidant species.

Nanoceria, or cerium oxide nanoparticles, have recently gained attention as an electrochemical sensor material due to their unique redox properties. Nanoceria can switch between Ce^3+^ and Ce^4+^ oxidation states, allowing them to act as both an oxidant and antioxidant depending on the surrounding environment [29]. Additionally, nanoceria have a high surface area, high catalytic activity, and excellent biocompatibility, making them an ideal candidate for sensing applications [30]. Some research groups already experimented with nanoceria in developing sensors for the evaluation of antioxidant compounds in wine [31,32]. Furthermore, this material can showcase pH-dependent biocatalytic properties [33] and has been proven to exhibit enzyme-like activities (i.e., peroxidase activity) [34], which can be exploited in the development of novel bioassays in medicine and dentistry.

In this study, we have developed a nanoceria-based electrochemical sensor for the detection of antioxidants in saliva. Commercial screen-printed carbon electrodes were modified by drop-casting ceria nanoparticle dispersion under the tenets of a lean project management strategy based on quality by design (QbD). In this sense, all research and development (R&D) pipeline was thoroughly planned, employing an experimental matrix obtained through a systematic design of experiments (DoE). The DoE is a statistical method that allows for the systematic evaluation of multiple parameters simultaneously, thereby reducing the number of experiments needed to obtain reliable results. We have optimized several parameters, including nanoceria concentration, drop-casting volume, and pH of the drop-casting solution, to investigate the influence of each factor on the analytical response. The goal of the present study is to demonstrate the feasibility of the analytical method and identify key optimization factors toward the development of a dedicated biosensing system capable of screening the AOC of saliva in a normal population, indicating whether it is in a healthy status or not. This study has the potential to lead to the development of portable and low-cost devices for point-of-care monitoring of antioxidant levels in human saliva, ultimately improving human health.

## 2. Materials and Methods

### 2.1. Materials and Reagents

The reagents used in the study were analytical-grade chemicals purchased from Sigma-Aldrich (St. Louis, MO, USA). The chemical reagents used included potassium hexacyanoferrate (III) (K_3_[Fe(CN)_6_]), potassium hexacyanoferrate (II) (K_4_[Fe(CN)_6_]), sodium chloride (NaCl), potassium chloride (KCl), sodium phosphate dibasic (Na_2_HPO_4_), potassium phosphate monobasic (KH_2_PO_4_), and ascorbic acid (AA). Cerium (IV) oxide NP (20 wt% colloidal dispersion in acetic acid 2.5 wt%, d = 30–60 nm), which was acquired from Merck (Darmstadt, Germany). SAT reagents were acquired from H&D s.r.l. Phosphate-buffered saline (PBS) solutions were prepared by dissolving 8 g of NaCl, 0.2 g of KCl, 1.44 g of Na_2_HPO_4_, and 0.245 g of KH_2_PO_4_ in 1 L of ultrapure deionized water (DI). The 5 mM [Fe(CN)_6_]^3−/4−^ solution used as a redox probe was prepared in 0.1 M PBS containing 0.1 M KCl. Screen-printed carbon electrodes (SPCEs) of model DRP-11L were purchased from Dropsens and consisted of a working carbon electrode, counter carbon electrode and Ag/AgCl reference electrode.

### 2.2. Electrochemical Measurements

Electrochemical measurements, comprising characterization tests with redox probe and calibration curves in the presence of the analyte, were performed with a portable potentiostat/galvanostat, PalmSens, EmStat4 Blue. PSTrace 5.9 software (PalmSens, Houten, The Netherlands) was used to run the experiments and automatically gather and store the experimental data. The electrochemical techniques used in the study were cyclic voltammetry (CV) and electrochemical impedance spectroscopy (EIS). CV and EIS were both employed in the characterization of the electrochemical sensors, while only CV was used to obtain the calibration curves. The parameters used for the CV characterization study were [−0.2–0.5 V] as the potential window, and 100 mV/s as the scan rate. The potential window was increased to [−0.2–1 V] for the calibration curves. EIS was also used to characterize the surface of the sensor during the development stages, and scans were run from 0.01 Hz to 10 kHz.

### 2.3. Sensor Modification

Nanoceria sensors were prepared from bare screen-printed carbon electrodes. A total of 35 electrodes were characterized via CV and EIS in the presence of a redox probe aiming the selection of the 24 electrodes that had the most similar behavior for the study. Once the batch of electrodes has been identified, a pretreatment was run on each of these electrodes to activate the carbon surface. SPCEs have a working electrode that consists of carbon flakes and a polymeric matrix. This creates a largely inert surface, which, however, can be activated with a simple treatment. About 50 µL of PBS has been dropped on the bare sensor surface, and a constant potential of 1.4 V has been applied for 300 s, after which the sensor has been dried. Following the pretreatment step, each electrode has been modified following the DoE experimental matrix, with a simple drop-casting procedure. After the drop-casting, the electrodes have been stored at room temperature for 1 day. Before each electrochemical test, the electrodes have been rinsed with deionized water (DI) and dried with nitrogen.

### 2.4. SAT Test

The SAT test has been chosen as the gold standard for the identification of the antioxidant capacity of liquid samples. A MiniSAT and a SAT50 kit have been purchased from H&D s.r.l. to perform the recovery tests. The procedure for the SAT test requires adding 40 µL of a reagent (R2) to a prefilled cuvette, acquiring the baseline, and then adding 10 µL of the test sample, after which the value of the concentration is returned by the SAT machine, expressed in mg/mol equivalents of ascorbic acid.

### 2.5. Quality-by-Design-Based Design of Experiments

Design of experiments (DoE) is a systematic and rigorous approach that is used to study a system and determine the correlation among its factors and the effects of each factor. It is a strategy that falls into the scope of refined industrial quality management systems. The quantification of the error that causes the variability in the system is based on the residual sum of squares. The DoE was used to optimize and evaluate the main effects of each factor, as well as their interactions and quadratic effects. In this work, a full-factorial experimental design based on a 2^3^ matrix was used: thus, three factors were selected, and two levels were tested for each factor. The factors herein studied were the nanoparticle concentration (A), drop-casting volume (B), and pH of the drop-casting solution (C), and they were all set as continuous independent variables. These factors were selected as critical key parameters in the experimental design. The nanoparticle concentration (A) can have an impact on the output current, as aggregates might form upon the use of overly concentrated solutions, which can block the diffusion of the antioxidants toward the electrode surface. The volume of the solution drop-casted on the electrode (B) can also impact the resulting currents since higher volume leads to bigger loading of particles per electrode when the concentration is fixed. This can have either beneficial or detrimental effects during assay; hence, it can saturate the surface and impair charge transfer. Lastly, the pH of the solution (C) has also been evaluated as a potentially relevant parameter, considering that the nanoparticles are produced and stored in an acidic solution (pH = 2.7), while dilutions are usually performed with DI water having a neutral pH. It is worth mentioning that these parameters were herein selected due to their critical effects on assay performance (i.e., quality); nevertheless, other factors not evaluated herein might also contribute to quality. In this regard, the strategy herein used was focused in achieving third-level technology readiness, which equals to proof of concepting.

## 3. Results and Discussion

**Figure 1:**
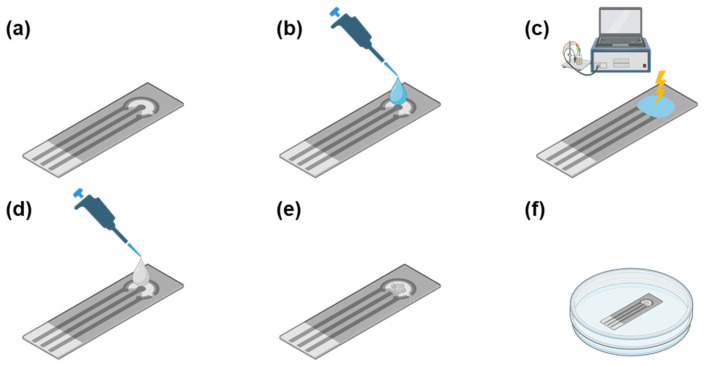
Nanoceria-modified SPCE development steps. (**a**) Bare screen-printed carbon electrode. (**b**) Drop-casting of a 50 µL droplet of PBS solution, pH = 7. (**c**) Surface activation via application of constant potential (E = 1.4 V) chronoamperometry for 300 s. (**d**) Drop-casting of 3 µL/5 µL of nanoceria solution depending on experimental design. (**e**) Nanoceria-coated screen-printed carbon electrode. (**f**) Storage at room temperature in dry conditions for 2 days.

The sensors were prepared following the procedure outlined in the Materials and Methods section and schematically summarized in Figure 1.

**Figure 2:**
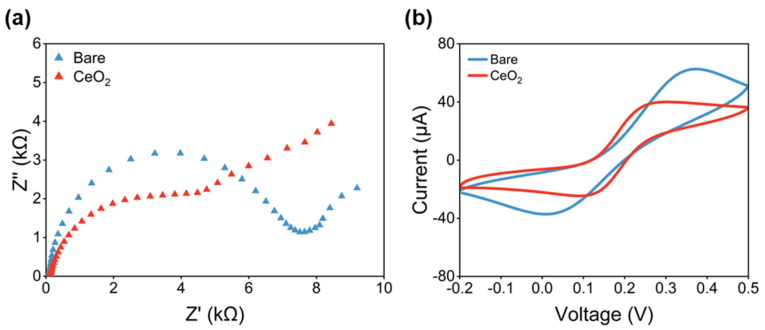
EIS and CV curves for electrode characterization studies in K_3_[Fe(CN)_6_]/K_4_[Fe(CN)_6_]. (**a**) EIS profile of bare (blue) and modified (red) screen-printed carbon electrode. (**b**) CV profile of bare (blue) and modified (red) screen-printed carbon electrode.

The experiments were run following the experimental matrix defined by the DoE in a randomized order to minimize experimental errors. Before executing the DoE, the electrodes were characterized by cyclic voltammetry and electrochemical impedance spectroscopy (EIS) to highlight the potential variability between carbon electrodes as provided by the manufacturer. The characterization studies were performed as described in the Materials and Methods section. Following the characterization studies, each electrode has been activated following a modified version of a surface activation procedure previously described in the literature [35]. Right after the electrode pretreatment has occurred, each electrode has been modified by drop-casting the nanoceria solution on the working electrode, following the DoE matrix. The results of the characterization in the K_3_[Fe(CN)_6_]/K_4_[Fe(CN)_6_] solution is reported in Figure 2.

The EIS in Figure 2a shows how the bare electrode (blue curve) has the characteristic shape of a bare carbon electrode, with a well-defined semicircle, that allows for the determination of the charge transfer resistance of the electrode. The modification with cerium oxide nanoparticles changes the profile of the EIS curve (red curve) introducing a plateau with a long diffusion tail, which is a characteristic of electrode surfaces modified with a nonconductive coating.

As seen in Figure 2, the predicted resistance to the charge transfer of the bare electrode, i.e., the diameter of the semicircle in the Nyquist plot, was about 8 kΩ. On the other hand, the predicted resistance to the charge transfer of the modified SPCEs was about 6 kΩ. According to the EIS and CV characterization, the semicircle profile could not be flawlessly seen in the Nyquist plots of the modified SPCE. This suggests that the electrooxidation/deposition may not be homogeneous, which has been previously suggested by other authors in studies about surface modification with nanostructured metal oxides [36].

**Figure 3:**
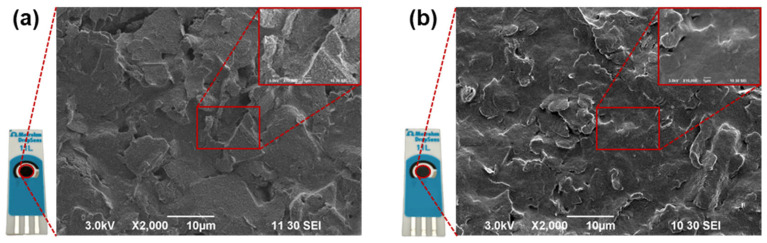
(**a**) SEM image of bare SPCE at 2000X magnification. Inset showing the bare electrode surface at 10,000× magnification. (**b**) SEM image of nanoceria-coated SPCE at 2000× magnification. Inset showing the nanoceria-coated SPCE surface at 10,000× magnification.

After the bare and modified SPCEs have been electrochemically characterized via EIS and CV, the surfaces of the two electrodes have been investigated via scanning electron microscopy (SEM) to confirm the successful deposition of nanoceria coating. The images in Figure 3a,b show the surfaces of the bare SPCE and nanoceria-modified SPCE, respectively. A white coating on the modified electrode surface is already visible to the naked eye, and the SEM image confirms the deposition of a nanoceria layer on top of the carbon surface (Figure 3b). The surface of SPCEs is generally composed of graphite flakes immersed in a polymeric matrix, as can be observed in Figure 3a. The nanoceria coating deposited on top of the rough carbon surface still presents a marked three-dimensional morphology, as the coating is not thick enough to smoothen the surface. The SEM images, however, confirm the successful deposition of the nanoceria coating, which was the aim of the microscopy investigation.

Upon confirming the successful surface modification of the electrode, the performance of the modified SPCE was tested in the detection of AA. The range of concentrations of ascorbic acid has been chosen based on the indications provided by the SAT test, a patented and clinically validated method for the evaluation of saliva AOC [37]. The test is based on the reduction of iron, similarly to how the ferric-reducing antioxidant power (FRAP) test works. The test has been validated on 70 saliva samples and provided the necessary information to draw physiological and pathological ranges for AOC, defined as equivalents of ascorbic acid. Healthy subjects are expected to have between 1 and 1.5 mM of AA equivalents. Values of AA equivalents lower than 1 mM and higher than 1.5 mM would signal the potential occurrence of inflammatory processes. Therefore, we decided to test our sensors in the 0.5–5 mM range to cover both physiological and pathological values.

**Figure 4:**
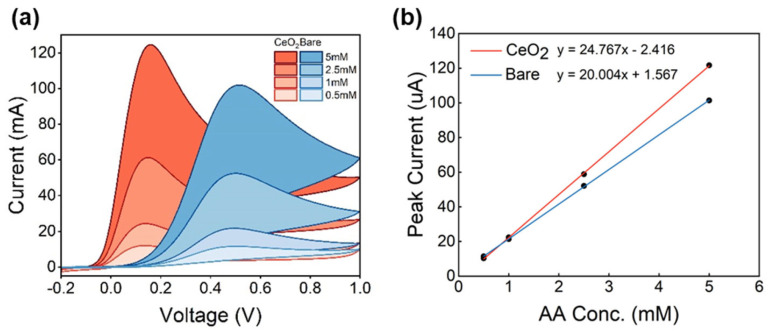
(**a**) CV curves of nanoceria-modified and bare SPCEs in the presence of AA. (**b**) Linear regression of bare and nanoceria-modified electrode responses in detecting AA. R^2^ of both fittings was 0.999.

As observed in Figure 4, AA electrooxidation occurred at a lower electric potential when analyzed with the modified SPCE, as opposed to the bare electrode. This hints that AA electrooxidation is thermodynamically favorable upon using the modified SPCE, which is in consonance with literature [30]. Moreover, the peak profile of AA detection was seemingly clearer upon the use of the modified SPCE, and the signal amplitude was higher. Considering that the higher the amplitude of an analytical signal, the lower the limit of detection (LoD) [38], it can be suggested that the modified SPCE not only evidences AA detection at lower electric potentials but also showcases better peak profile and higher amplitudes, which can imply lower LODs. As such, the modified SPCE exhibited adequate linearity, as well as adequate LODs and signal amplitude for the purpose they were herein designed to attend to; we, therefore, selected the LOD and signal amplitude as the two main parameters for analytical optimization.

Aiming to investigate the influence of the selected parameters on the analytical performance of the modified SPCE, a full 2^3^ DoE was performed and subjected to desirability functions. Thence, the experimental matrix was constituted of 2 levels and 3 factors, as coded in the Materials and Methods section and reported in Table 1. The choice of the different levels for each factor is based on previous works in the context of the development of nanoceria-based sensors [31,32]. The concentration of the nanoceria particle solution as purchased is 20 wt%, and most of the works in the literature operate a 1/10 dilution; thus, we wanted to investigate the effect that the particle density has on the final nanoceria coating. Similarly, volumes of both 2 µL and 5 µL have been reported in previous studies [31,32]; thus, we deemed that the drop-casting volume was another parameter that needed to be systematically investigated. As for the pH of the solution, the cerium oxide nanoparticle dispersion has an acidic pH of 2.7; thus, a dilution was performed with both DI water at pH of 7 and with DI water and 2.5% acetic acid at a pH of 2.7.

All experiments were performed in triplicates and consisted of individual assaying of 4-point calibration curves, which were also performed in triplicates. Moreover, each calibration curve was used to calculate the concentration of the antioxidant standard AA in a recovery test, whose results were confronted by a standard AOC test, namely SAT.

The recovery test herein used is based on the analysis of observed versus expected values obtained from the solutions of known concentrations, which are assayed with the gold standard method (i.e., SAT) and the proposed method (the nanoceria sensor). This test evaluates the accuracy of the method and allows the collection of data about how reliably it can be used to adequately measure analyte concentrations. Due to the sheer volume of data, the mean of the individually performed triplicates was calculated and herein used as input for the dependent variables. For the purpose of investigating the factors that influence the most on the sensitivity of the modified SPCE, the dependent variables herein elected to undergo the desirability study were the LoD and signal amplitude increment of AA oxidation, which is herein used as the analytic signal and was labeled ∆Ip.

The LoD is a parameter of sensitivity, being defined by the lowest detectable quantity of an analyte in a sample. The LoD is calculated by the product of a constant factor (i.e., 3.3) and the quotient of the standard deviation and the slope of the calibration curve. Hence, the lower the value of the LoD is, the more likely it is to measure minute concentrations of analytes in a sample; thus, this parameter follows, therefore, an inversely proportional distribution with sensibility. The experimental matrix is showcased in Table 2.

As observed in Table 2, the LoD ranged from 0.1147 to 0.3528 mM, while the recoveries varied from 80% to 121.1%. Overall, the results were within the expected ranges for analytical applications targeting the measurement of AOC in saliva. Regarding the recoveries, all sensors except #2 showcased values within the optimal range. Literature states that optimal values for recovery assays range from 70 to 120% with about 20% of relative standard deviation (RSD) [39].

**Figure 5:**
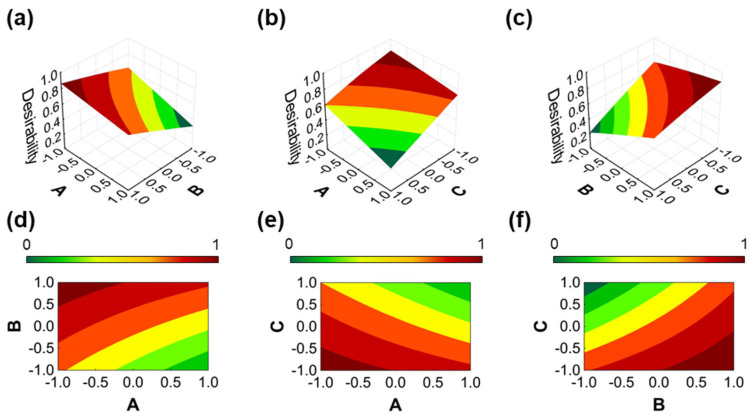
Desirability profile of LoD and ∆Ip. Both dependent variables were normalized between 0 and +1, while LoD values were normalized and rescaled in inversed order, hence the inversely proportional nature of LoD and sensitivity. A: NP loading; B: drop-casting volume; C: pH of drop-casting solution. (**a**) 3D plots showing the combined effect of NP loading and drop-casting volume on the LoD. (**b**) 3D plots showing the combined effect of NP loading and pH of drop-casting solution on the LoD. (**c**) 3D plots showing the combined effect of drop-casting volume and pH of drop-casting solution on the LoD. (**d**) Contour plot of LoD as a function of NP loading and drop-casting volume. (**e**) Contour plot of LoD as a function of NP loading and pH of drop-casting solution. (**f**) Contour plot of LoD as a function of drop-casting volume and pH of drop-casting solution.

Following the initial assessment of the data, the LoD and ∆Ip values were subjected to desirability functions. These functions allow the simultaneous evaluation of the dependent variables in order to create a predictive model of an ideal optimized response [40]. This treatment was herein performed, hence the relevance in investigating how the variables contribute to the improvement of the analytical response. The results are showcased in Figure 5.

The results of the desirability functions suggest that factor A, i.e., NP loading, negatively correlates with the descriptors of analytical sensitivity. This can be seen in Figure 5a,b,d,e, wherein the desirability surface exhibited minimum values (color-coded in green) when A was at the highest level. Conversely, factor B, i.e., the drop-casting volume, positively correlates. This interpretation is based on the observation of Figure 5a,c,d,f, wherein the desirability was at higher values (color-coded in red) when factor B was at the highest level. As for variable C, i.e., the pH of the drop-cast solution, it negatively correlates with the analytical descriptors. This can be evaluated in Figure 5b,c,e,f, wherein the desirability was lower (color-coded in green) when factor C was at the highest level.

The use of objective functions to predict likelihood distributions and even optimal parameters in processes has been previously explored in the literature [41]. This method involves the conversion of the estimated response into an individual composite function, which is based on several individual desirability functions [42]. The surface model yielded by the application of this mathematical treatment allows the extrapolation of experimental observations and permits inferences to be reliably made about behaviors outside the limits of experimental designs, such as the one herein used.

The effect of the surface modifier concentration in drop-casting solutions has been explored in electrochemical sensing [43,44]. It is known that the dielectric nature of some modifiers may impair charge transfer on the working electrode surface, thereby hindering the analytical performance [45]. Conversely, the concentrated solutions of conductive materials can also lead to poor performance [46]. Such seemingly paradoxical effect is likely attributable to irregular surface deposition. In fact, this was observed in other works detailing the use of nanostructured metal oxides in crafting redox sensors such as the one herein investigated [47,48]. In our desirability study, it is suggested that the increase in the NP loading in the drop-cast solution leads to a drop in performance, which could be possibly attributed to the aforementioned inhomogeneous deposition.

Drop-casting is a widely used protocol for surface modification in electrochemical sensing due to the easy execution and straightforwardness [44]. However, the volume to be added needs to be adjusted to the dimensions of the electrode set, as well as take into consideration the wettability of the transducer [44]. Moreover, the adequate surface is dependent on intrinsic fluid parameters such as capillarity [49]. In our work, it was noticed that diminished drop-cast volumes were not only harder to dispense on the surface but also likely to compromise the analytical performance, as suggested in the desirability study. This could be possibly attributed to inhomogeneous surface coverage during the surface modification step [44].

Although not directly investigated in this report, the nanozyme activity of nanoceria has been described to be influenced by the particle size [34]. We set the particle size in this report between 30 and 60 nm, which was the range offered by the manufacturer. Nevertheless, these particles can be synthesized in-house, and their size can be accordingly tailored. Concerning the catalytic activity, it has been demonstrated elsewhere that an increase in the nanoparticle size from 7 to 15 nm leads to a decrease in the activity by a factor of 1.2 [50]. Moreover, it has been suggested that small particle sizes (i.e., 5 nm) may have more than 30 times the catalytic power of particles sized 10 nm [51]. In this sense, the particle size can be also considered a parameter that is critical to performance, and it will be further investigated in future studies.

Regarding the influence of pH in the analytical performance, it is known that the acidity of the solution plays a major role in the capacity of metals to attain charge [52]. This characteristic has deep implications in electrocatalysis [53]. In general, transition metals behave as Lewis acids due to vacant d-orbitals. This allows charge transfer processes to be more thermodynamically feasible and is the major interpretation for the electrocatalytic effect [54]. It is worth mentioning that metals may differently behave upon attaining charge, which can diminish or even turn electrocatalysis unfeasible. Another point to be considered is the salivary pH, which can also affect the assay. The salivary pH may drop to about 6.3 in physiological conditions, which can influence the output of the test. Nevertheless, it must be mentioned that most salivary antioxidant capacity tests are prone to this interference, hence the inherent correlation between pH and redox processes. In any case, putative pH interferences will be thoroughly evaluated in future outreaches by our group. To all accounts, the use of more acidic solutions may possibly allow the nanoparticles to remain in their uncharged state, which not only contributes to the maintenance of their electrocatalytic power but also can potentially assist in their homogenous deposition on the carbon surface.

## 4. Conclusions

This study reported a detailed and systematic analysis of the process of electrode functionalization with cerium oxide nanoparticles. The study confirmed the biomimetic properties of the nanozyme in the context of the detection of a common antioxidant, ascorbic acid. The significant shift in the peak potential (~400 mV) during the cyclic voltammetry analysis proved the electrocatalytic properties of nanoceria, pointing at promising applications of this nanomaterial in the development of sensors and biosensors for AOC evaluation. The LoDs ranged from 0.1147 to 0.3528 mM, while the recoveries varied from 80% to 121.1%, being, therefore, comparable with those of the gold standard SAT test, whose recovery value was 96.3%. The QbD-based DoE approach used in the study allowed us to identify important correlations between the different factors characterizing the functionalization process and the analytical parameters of interest. The results of the desirability functions suggest that nanoparticle loading and the pH of the drop-casting solution negatively correlate with the descriptors of analytical sensitivity, while the drop-casting volume positively correlates. We also validated the applicability of the developed sensors in detecting AOC within the physiological and pathological ranges expressed in saliva by performing recovery studies against the gold standard for saliva AOC evaluation, the SAT test. While the recovery values where within acceptable ranges as defined in the literature, future studies will be focused on improving the analytical performance of the sensors and recovery values through an optimization of the functionalization process based on the learnings of the present study. Lastly, the research team will include in future studies tests of the sensors in real saliva samples from a healthy population to demonstrate the applicability in clinical settings.

## Figures and Tables

**Table 1:** Coded factors and levels.

	Minimum Value (−1)	Maximum Value (+1)
**NP loading (A)**	0.5%	2%
**Drop-casting volume (B)**	3 µL	5 µL
**pH of drop-casting solution (C)**	2.7	7

**Table 2:** Experimental matrix in standard order. LoD values were normalized and rescaled in inversed order between 0 and 1 (LoD norm). This was performed since LoD and sensitivity are inversely proportional. SAT recovery value against theoretical target value was 96.3%.

Exp	A	B	C	LoD (mM)	Recovery	∆Ip (µA)	LoD Norm	∆Ip
#1	−1	−1	−1	0.1489	111.0%	8.937	0.8564	0.8756
#2	1	−1	−1	0.1235	121.1%	−15.65	0.9630	0.1726
#3	−1	1	−1	0.1711	111.1%	10.54	0.7631	0.9215
#4	1	1	−1	0.1489	80.0%	13.29	0.8564	1
#5	−1	−1	1	0.1436	121.1%	−21.68	0.8786	0
#6	1	−1	1	0.3528	102.5%	−14.05	0	0.2183
#7	−1	1	1	0.1147	116.2%	5.026	1	0.7638
#8	1	1	1	0.1586	101.2%	−13.40	0.8156	0.2369

Exp: experiment run; A: NP loading; B: drop-casting volume; C: pH of drop-casting solution; LoD: limit of detection, ∆Ip: variation in electric signal amplitude of faradaic processes (i.e., peaks); LoD norm: normalized values of LoD between 0 and 1.

## Data Availability

The data presented in this study are available on request from the corresponding author.

## Supplementary Materials

The following supporting information can be downloaded at: https://www.mdpi.com/article/10.3390/antiox12051120/s1, Figure S1: Validation against state-of-the-art SAT test.
