# Supplementary material for: Quality-by-Design R&D of a Novel Nanozyme-Based Sensor for Saliva Antioxidant Capacity Evaluation
Source: Antioxidants (Basel). 2023 May 18;12(5):1120. doi: 10.3390/antiox12051120 (PMC10215665; doi:10.3390/antiox12051120)
Supplement: Supplementary file 1 [file antioxidants-12-01120-s001.zip › antioxidants-2405755-supplementary.pdf]

Quality by Design R&D of a Novel Nanozyme Based Sensor for Saliva Antioxidant Capacity Evaluation—Supplementary Information

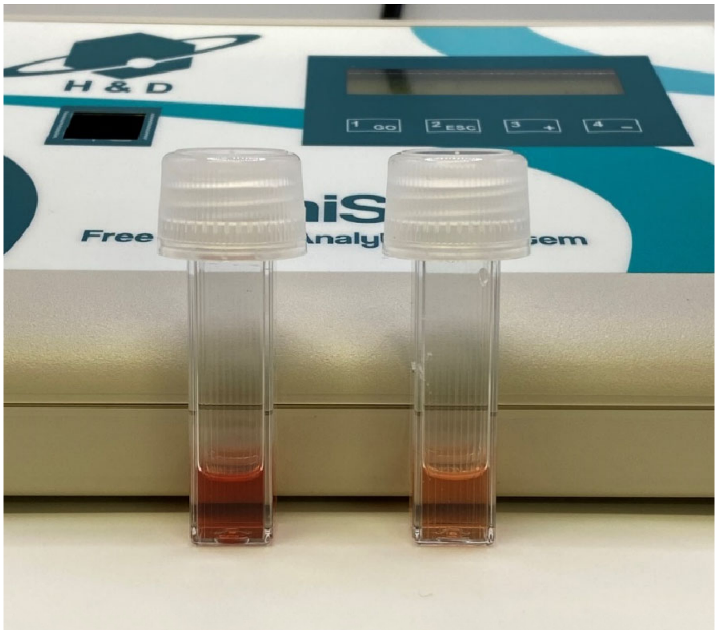

Figure S1. Validation against state-of-the-art SAT test. Benchtop spectrophotometer (MiniSAT) in the background, and color shift induced by the addition of ascorbic acid to the cuvette filled with reagents in the foreground.
